# Antibody‐Conjugated Magnetic Nanoparticle Therapy for Inhibiting T‐Cell Mediated Inflammation

**DOI:** 10.1002/advs.202307148

**Published:** 2023-12-31

**Authors:** Mahbub Hasan, Jong‐Gu Choi, Hafeza Akter, Hasung Kang, Meejung Ahn, Sang‐Suk Lee

**Affiliations:** ^1^ Department of Digital Healthcare Sangji University Wonju 26339 South Korea; ^2^ Department of Biochemistry and Molecular Biology Life Science Faculty Bangabandhu Sheikh Mujibur Rahman Science and Technology University Gopalganj 8100 Bangladesh; ^3^ Department of Medicine College of Medicine Seoul National University Seoul 08826 South Korea; ^4^ Department of Animal Science College of Life Science Sangji University Wonju 26339 South Korea

**Keywords:** anti‐CD3, immunotherapy, inflammation, magnetic nanoparticles

## Abstract

Tolerance induction is critical for mitigating T cell‐mediated inflammation. Treatments based on anti‐CD3 monoclonal antibody (mAb) play a pivotal role in inducing such tolerance. Anti‐CD3 mAb conjugated with dextran‐coated magnetic nanoparticles (MNPs) may induce inflammatory tolerance is posited. MNPs conjugated with anti‐CD3 mAb (Ab‐MNPs) are characterized using transmission and scanning electron microscopy, and their distribution is assessed using a nanoparticle tracking analyzer. Compared to MNPs, 90% of Ab‐MNPs increased in size from 54.7 ± 0.5 to 71.7 ± 2.7 nm. The in vitro and in vivo studies confirmed the therapeutic material as nontoxic and biocompatible. Mice are administered various dosages of Ab‐MNPs before receiving concanavalin‐A (ConA), an inflammation inducer. Preadministration of Ab‐MNPs, as opposed to MNPs or anti‐CD3 mAb alone, significantly reduced the serum levels of interferon‐γ and interleukin‐6 in ConA‐treated mice. Additionally, the transdermal stamp patch as an effective delivery system for Ab‐MNPs is validated. This study demonstrates the utility of the Ab‐MNP complex in pathologies associated with T cell‐mediated hyperinflammation, such as organ transplantation and COVID‐19.

## Graphical abstract

Treatments based on the glutaraldehyde‐based conjugation of anti‐CD3 antibodies (Ab) with dextran‐coated magnetic nanoparticles (MNPs) play a pivotal role in tolerance induction. Preadministration of Ab‐MNPs via intravenous administration and intradermal stamp patch‐based delivery inhibited concanavalin A (ConA)‐induced cytokine (IFN‐γ, IL‐6) hypersecretion. This study demonstrates the utility of the Ab‐MNP complex in pathologies associated with T cell‐mediated hyperinflammation, such as COVID‐19.

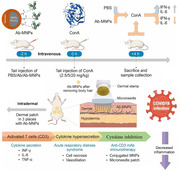

## 1 Introduction

T‐cell function relies on the activation of the multi‐subunit apparatus cluster of differentiation 3 (CD3) complex. This complex and αβ T‐cell receptor (TCR) form the TCR–CD3 complex, which triggers the activation of T cells. Key components of the CD3 complex include single units of αβTCR, CD3εγ, CD3εδ, and CD3ζζ dimers.^[^ ^1^ ^]^ The murine anti‐CD3 monoclonal antibody (mAb), OKT3, was the first mAb‐based immunosuppressant drug used to reduce organ rejection in patients receiving kidney, liver, or heart transplants. However, the clinical development of OKT3 was halted owing to its adverse effects. In contrast, the mouse‐specific mAb clone 145‐2C11 did not induce such toxicity in a murine model.^[^ ^2^ ^]^

The fundamental mechanism of anti‐CD3 mAb treatment involves inducing tolerance through the shedding or internalization of the TCR‐CD3 complex, rendering T cells unresponsive to proinflammatory antigens. Moreover, signaling induced by anti‐CD3 mAb through the TCR‐CD3 complex can trigger T cell apoptosis. The resulting apoptotic T cells, along with the macrophages detecting them, release transforming growth factor‐β (TGF‐β), further promoting tolerance.^[^ ^3^ ^]^ Anti‐CD3 mAb can bind to the Fc receptors of antigen‐presenting cells (APCs) and the TCR‐CD3 complex.^[^ ^3^ ^]^ Therefore, preventing Fc‐mediated interactions with APCs is necessary to stop the activation of T cells and the subsequent increase in inflammatory cytokine levels. Humanized anti‐CD3 mAb (HuM291) was designed to bind specifically to the CD3ε chain of the TCR, avoiding Fc‐mediated binding and complement fixation. Based on preclinical and clinical research data, HuM291 effectively depletes circulatory T cells.^[^ ^4^ ^]^ However, after phase II/III clinical trials, further development of HuM291 was discontinued owing to concerns regarding its efficacy and safety profiles.

Magnetic nanoparticles (MNPs) have been extensively used in imaging, drug delivery, and therapeutics. The surface modification and dosage of MNPs determine their in vivo biodistribution patterns and toxicities. Size is a crucial factor for nanoparticle bioavailability. MNPs larger than 200 nm accumulate in the spleen, while those smaller than 10 nm are easily eliminated through renal clearance. Therefore, based on the size criterion,^[^ ^5^ ^]^ MNPs ranging between 10 and 100 nm are optimum for theragnostics. Superparamagnetic nanoparticles do not tend to self‐agglomerate and exhibit magnetic properties only under an external magnetic field. The outer surface of MNPs can be functionalized with different molecules, including inorganic molecules, small organic molecules, and polymeric materials.^[^ ^6^ ^]^ Dextran, a complex branched polymer composed of glucose‐containing chains of varying lengths (3–2000 kDa), is highly biocompatible, biodegradable, and water‐soluble.^[^ ^7^ ^]^ It allows the efficient conjugation of ligands with functional groups, for example, anhydrides, amines, hydroxyls, carboxylic acids, thiols, and epoxides on the surface of nanoparticles, enabling the synthesis of diverse antibody (Ab)‐conjugated nanoparticles or Ab‐drug conjugates.^[^ ^8^ ^]^ Glutaraldehyde‐based Ab conjugation with nanoparticles containing amine functional groups on their surface allows for specific antigen binding.^[^ ^9^ ^]^ The functional groups enable the covalent binding of proteins, Abs, or other molecules to the surface of the MNPs.^[^ ^10^ ^]^ Glutaraldehyde acts as a crosslinker during the covalent binding process. MNPs conjugated with anti‐CD3 mAb (Ab‐MNPs) were designed to inhibit CD3–TCR complex formation and induce immune tolerance.

In this study, we synthesized and characterized a novel Ab‐MNP complex. Additionally, we aimed to confirm this complex's anti‐inflammatory efficacy through intravenous administration and intradermal stamp patch‐based delivery using a mouse model of concanavalin A (ConA)‐induced inflammation. We became interested in dermal stamps owing to their extensive use in the cosmetic industry and their potential application in transdermal drug delivery.^[^ ^11^ ^]^ We aimed to develop a valuable therapeutic strategy for clinical conditions such as severe acute respiratory syndrome coronavirus 2 (SARS‐CoV‐2) infections associated with enhanced proinflammatory states leading to multiorgan failure,^[^ ^12^ ^]^ organ rejection after organ transplantation, and autoimmune diseases.

## 2 Results and Discussion

### 2.1 Validation of Ab‐MNP Conjugation

**Figure 1:**
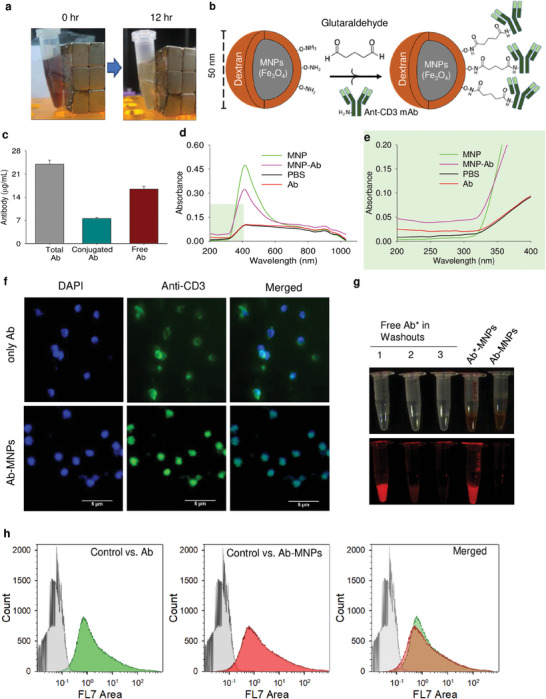
Conjugation of anti‐CD3 monoclonal antibodies (mAb) with dextran‐coated magnetic nanoparticles (MNPs). a) 12 h separation of dextran‐coated MNPs from the dispersive solution using a permanent magnet. b) Scheme of glutaraldehyde‐based conjugation. c) Measurement of the conjugated and unconjugated mAbs by BCA assay. d,e) Spectrophotometric confirmation of distinct features of anti‐CD3 monoclonal antibody‐conjugated MNPs (Ab‐MNPs). f) Immunohistochemical analysis of the antigen‐binding properties of the mAbs (Ab and Ab‐MNPs) against CD3 of the splenocytes. The CD3ε receptors on the cell surface were stained using fluorescein isothiocyanate (FITC; green), and nuclei were stained with 4′,6‐diamidino‐2‐phenylindole, dilactate (DAPI; blue). g) Fluorescent images of conjugated MNP‐Ab. MNP and Novaflour700‐tagged anti‐CD3 antibodies were conjugated. The free/unconjugated antibodies were removed by buffer separation using an external magnet. The fluorescent intensities were measured in three consecutive washouts and the conjugated MNP‐Ab‐Flour700 solution. h) Lymphocytes were gated based on location in the FS area versus the SS area. The histograms of the stained CD3‐positive cells were plotted either only Novaflour700‐tagged anti‐CD3 or, anti‐CD3 conjugated MNPs. Unstained cells were used as the control sample.

Conjugation of anti‐CD3 monoclonal Abs with dextran‐coated MNPs (Ab‐MNPs) involves a two‐step reaction. In this study, buffer exchange was performed by placing the MNP solution on a magnet for 12 h (**Figure** **1**a). Supernatants were carefully discarded using a micropipette. Glutaraldehyde is known to act as a linker, forming two amide bonds: one with the amine functional groups in MNPs and another with the amino acids within Abs (Figure 1b). The conjugation of Abs with MNPs was validated through various methods, including bicinchoninic acid (BCA) protein assay, spectrophotometry, and immunocytochemistry. After reacting Abs (24 µg mL^−1^) with glutaraldehyde‐linked MNPs, the mixture was washed three times to remove unbound Abs. The antibody concentration was measured in each washout buffer collected, starting at 16 µg mL^−1^ in the first washout buffer and gradually reducing to zero in subsequent washes. Consequently, 8 µg (33.3%) of the Abs was successfully conjugated with 1 mL of MNPs (1 mg mL^−1^) (Figure 1c).

The amount of Ab in 100 µL of Ab‐MNPs was ≈0.8 µg. The UV–Vis absorption spectra (200–1000 nm) of the MNPs (1 mg mL^−1^), Abs (10 µg mL^−1^), and Ab‐MNPs revealed distinct patterns (Figure 1d). The absorbance of Ab‐MNPs at 280 nm (protein absorbance) confirmed the presence of Abs in the conjugates. In a more extensive view ≈280 nm, the spectrum showed that MNPs have a lower absorbance than Ab‐MNPs (Figure 1e). Splenocytes collected from the spleen contained lymphocytes, macrophages, and dendritic cells. Among the lymphocytes, the percentage of T and B cells expressing CD3ε was ≈41% and 44%, respectively.^[^ ^13^ ^]^ The splenocytes were stained with anti‐CD3 or Ab‐MNPs after mitogenic stimulation with ConA. The expression of CD3ε on the cell surface was assessed by staining with fluorescein isothiocyanate (FITC)‐labeled secondary Abs. The nuclei were stained with 4′,6‐diamidino‐2‐phenylindole dilactate (DAPI) (Figure 1f). We further conjugated MNPs with Novaflour700‐tagged anti‐CD3, and the fluorescence Ab‐conjugated MNPs were utilized for in vivo biocompatibility and flow cytometry assay to validate the antigen binding ability by flow cytometry. The unbound antibodies were removed by buffer separation using an external magnet. The fluorescent intensities were measured in three consecutive washouts and the conjugated MNP‐Ab‐Flour700 solution (Figure 1g). Splenocytes collected from freshly dissected mice were stained fluorescent‐tagged anti‐CD3 or MNP‐conjugated anti‐CD3. The unstained splenocytes were used as control samples. First, lymphocytes were selected using FS area/SS area and FS height/SS area scatter plots. Two hundred thousand cells were gated as lymphocytes, representing 53% of total splenocytes (Figure S1, Supporting Information). Flow cytometry data revealed that the CD3‐positive cells selected by both Ab and Ab‐MNPs were not significantly different (Figure 1h). The results showed that our Ab‐MNPs conjugate can efficiently bind to CD3ε expressed on the cell surface. This conjugation preserves the Fab portion of the Abs, which is available for tying with antigens.^[^ ^14^ ^]^

### 2.2 Characterization of MNPs and Ab‐MNPs Using Scanning Electron Microscopy

**Figure 2:**
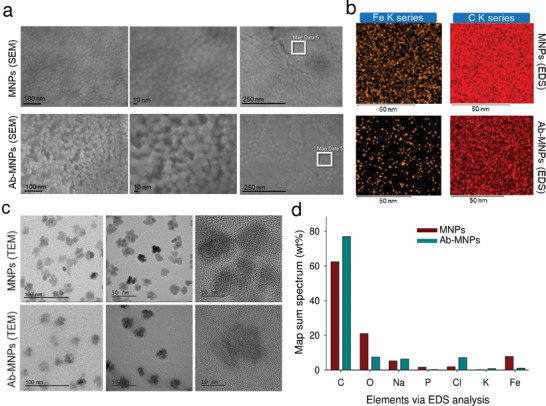
Characterization of MNPs and anti‐CD3 monoclonal antibody‐conjugated MNPs (Ab‐MNPs). Elemental analysis of the surface of MNPs and Ab‐MNPs. a) Scanning electron microscopy (SEM) 3D images of nanoparticles. b) Energy dispersive X‐ray spectroscopy (EDS)‐layered images captured in a localized area. The elements were measured by default. Representative Fe (K) and C (K) images are shown. c) Transmission electron microscopy (TEM) 2D images of nanoparticles at different magnifications. d) The EDS data revealed the map sum spectra of different elements. The elements are shown as wt.% of total sum spectra.

The samples were analyzed using a field‐emission scanning electron microscope (SEM; JSM‐7900F; JEOL Ltd., Tokyo, Japan) to monitor the surface of the particles. The particles were well separated, and the size of the Ab‐MNPs increased compared with MNPs, as shown in **Figure** **2**a. A transmission electron microscope (TEM, JEM‐F200; JEOL Ltd.) was used to obtain magnified images. The nanoparticles dissolved in phosphate‐buffered saline (PBS) were layered on a TEM grid, dried in open air, and visualized using TEM. Figure 2b presents images of MNPs and Ab‐MNPs at varying magnifications, revealing well‐dispersed particles in the solution. Compared to the corresponding image of not conjugated MNPs, the Ab‐conjugated MNPs had a distinctive outer layer (Figure 2b; 50 nm scale) because of the Abs attached to their surfaces. The image of the Ab‐conjugated MNPs also revealed an increase in the hydrodynamic diameter of Ab‐MNPs.

Furthermore, energy‐dispersive X‐ray spectroscopy (EDS) was used to analyze the surface elemental composition of localized areas densely populated with nanoparticles (Figure 2c). The elements selected by default (Fe, C, and O) by the EDS software were the major constituents of MNPs (Fe_3_O_4_), dextran, and Abs. The density of Ab‐MNPs was lower than that of MNPs, as shown in Figure 2c,d. Therefore, a decrease in the Fe and O map sum spectra (wt.%) supported the idea that Ab‐MNPs are less dense than MNPs. However, even with a reduction in Fe and O, a subsequent increase in C (wt.%) indicates the conjugation of Abs to the nanoparticle surface (Figure 2d).

### 2.3 Comparisons of Nanoparticles Based on Size Distribution and Diffusion Coefficient

**Figure 3:**
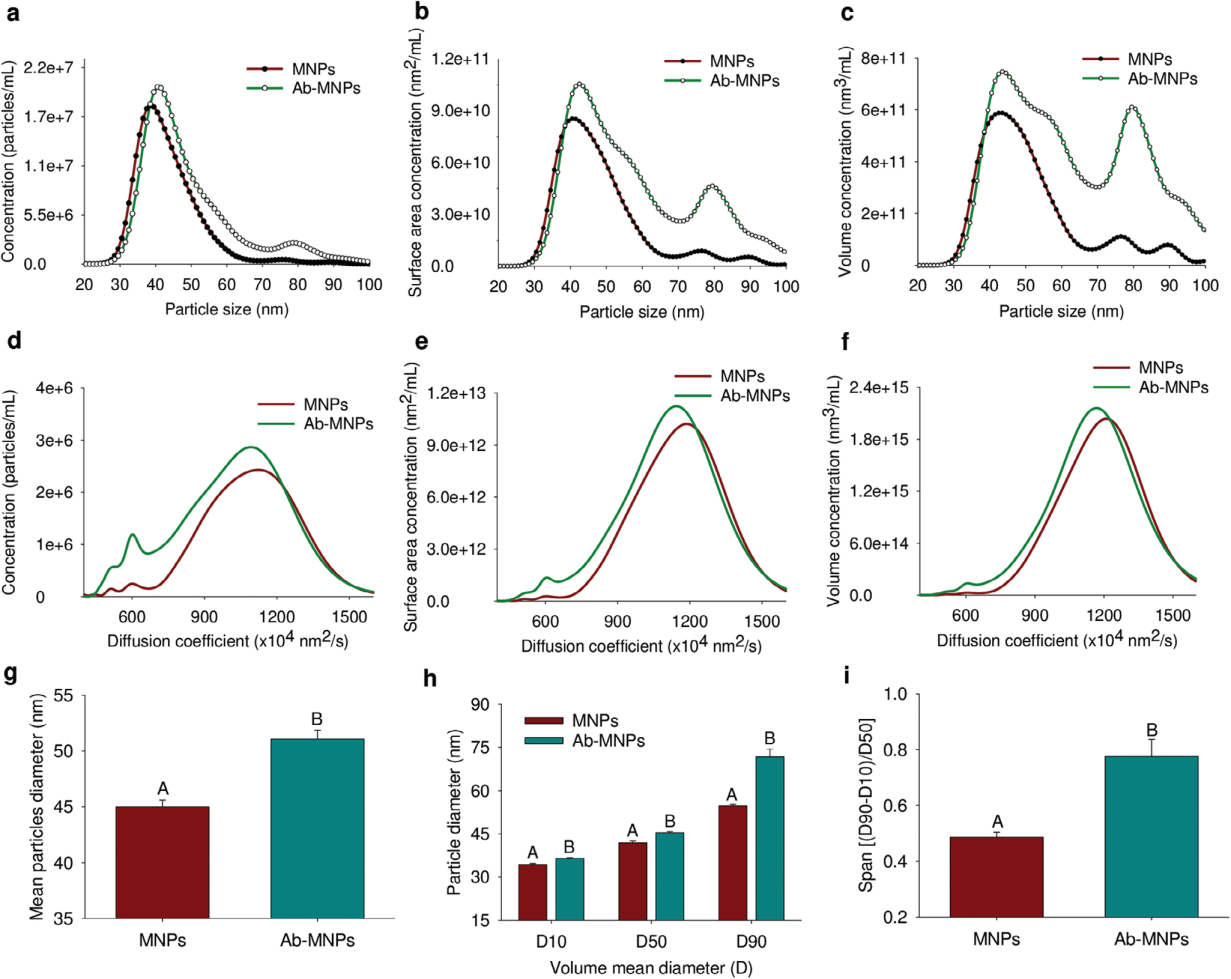
Comparison of magnetic nanoparticles (MNPs) and anti‐CD3 monoclonal antibody‐conjugated MNPs (Ab‐MNPs) based on size and diffusion coefficients. Size distribution of MNPs and Ab‐MNPs by diameter and mean volume diameter. The concentration (particles/mL), surface area concentration (nm^2^ mL^−1^), and volume concentration (nm^3^ mL^−1^) of MNPs and Ab‐MNPs are based on their particle size a–c) and diffusion coefficient d–f). Video data of nanoparticle tracking analysis measurements were collected for 30 s and repeated five times for each sample. Average observed diameter of MNPs and Ab‐MNPs g) and the distribution of nanoparticle size evaluated by diameter (D10, D50, and D90) h). Particle size distribution based on the span value (D90‐D10)/D50 i). The lower spun value indicates a more homogenous particle size. However, span values less than 1 are regarded as a homogenous distribution. The data are shown as mean ± standard error of the mean (*n* = 5 samples per group). *^A, B^* Different letters indicate significant differences between groups (*p* < 0.05 vs only MNPs).

Nanoparticle tracking analysis (NTA) measures the Brownian motion of nanoparticles by analyzing the movement of laser–beam–illuminated particles. The diffusion coefficient reflects the random movement of nanoparticles. Here, the concentration (particles/mL), volume concentration (nm^3^ mL^−1^), and surface area concentration (nm^2^ mL^−1^) were measured based on the particle size (**Figure** **3**a–c) and diffusion coefficient (Figure 3d–f). The diffusion coefficient correlated with the hydrodynamic diameter of the nanoparticles. As the size of the nanoparticles decreases, the diffusion activation energy increases, thereby increasing the diffusion coefficient.^[^ ^15^ ^]^ In this study, the diffusion coefficients of larger particles decreased as the diffusion activation energy decreased. The concentration of larger particles increased upon conjugation with Abs, as indicated by the shift to the right in the size versus concentration curve (Figure 3a). Concurrently, the diffusion coefficient versus concentration curve shifted to the left (Figure 3d).

Similarly, the increased concentration of larger particles represented by Ab‐MNPs increased the volume and surface area concentrations (Figure 3b,c). Simultaneously, the volume and surface area concentrations of Ab‐MNPs with lower diffusion coefficients increased (Figure 3e,f). Furthermore, the average drift velocities of MNPs and Ab‐MNPs on both *x*‐ and *y*‐axes were evaluated.^[^ ^16^ ^]^ However, the difference between them was insignificant.

The average observed diameter of MNPs and Ab‐MNPs was 45.0 and 51.06 nm, respectively (Figure 3g). Nanoparticle size distribution can also be evaluated using the volume mean diameter (D_V_) or D10, D50, and D90. The distribution parameters were determined using laser diffraction, and the size of the nanoparticles in the cumulative volume distribution was characterized. D50, the median diameter, indicates that half of the nanoparticles are below this value, and half are larger. Similarly, D10 and D90 show the corresponding size distributions of the nanoparticles. The span value (D90–D10)/D50 was also measured.^[^ ^17^ ^]^ The D50, D10, and D90 values were significantly greater for Ab‐MNPs than for MNPs (Figure 3h). This finding suggests an increase in hydrodynamic diameter for both smaller (<40 nm) and larger (>40 nm) nanoparticles. Furthermore, in Ab‐MNPs, 90% of the particles increased in size from 51 to 71 nm. Although the span values were higher for Ab‐MNPs than for MNPs (0.49 vs 0.78), they remained below 1.0, indicating a unified size distribution (Figure 3i).

### 2.4 Toxicity and Biocompatibility Evaluation

**Figure 4:**
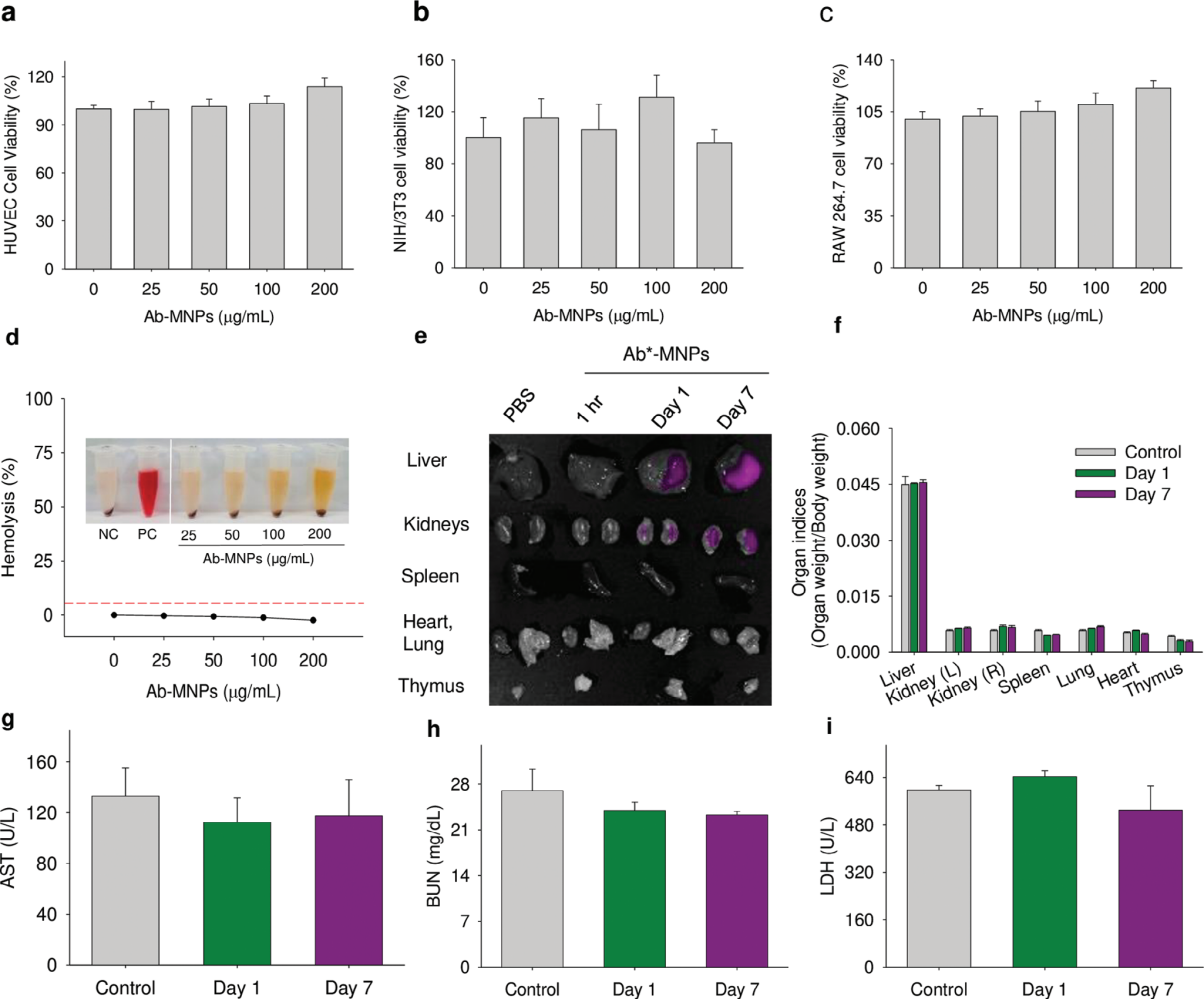
Toxicity and biocompatibility of Ab‐MNPs. In vitro CCK‐8 assay of cell viability was evaluated in a) human HUVEC, b) mouse NIH/3T3 fibroblast, and c) macrophage RAW246.7 cell lines. Cells were treated with different concentrations of Ab‐MNPs for 24 h. Data are presented as mean ± standard error of the mean (*n* = 5). d) A relative hemolysis rate after 1 h incubation of different concentrations of Ab‐MNPs in mouse RBCs at 37 °C. Inside images of treated samples, negative control (NC), and positive control (PC) after centrifugation at 10,000 rpm for 5 min. A large amount of hemoglobin (red color) was observed only in the positive control sample. e) Ex vivo fluorescence analysis after intravenous treatment of Ab*‐MNPs (*Fluorescence‐tagged anti‐CD3 mAb) in mice. Samples were collected after 1 h, 1 day, and 7 days post‐treatment. Represented images of three mice/group. Mice were further treated with Ab‐MNPs at the treatment dose (5 mg kg^−1^), and in vivo toxicity was measured. Organ indices f) and serum biochemical markers, including AST g), BUN h), and LDH i), were measured by a clinical sample analyzer. Samples were presented as mean ± standard error of the mean. Different letters indicate significant differences between groups (*p* < 0.05).

The toxicity and biocompatibility data evaluate the health impacts of the nanoparticles. The in vitro and in vivo toxicity studies are performed using different methods. The most common in vitro methods include cell proliferation, viability, and metabolic activity.^[^ ^18^ ^]^ Additionally, oxidative stress, genotoxicity, apoptosis, necrosis, and immunotoxicity are routinely analyzed. The in vivo toxicity tests comprising the biocompatibility evaluation include the effects of the nanoparticles on the organ and immune system.^[^ ^19^ ^]^ The initial observations typically include body weight and behavioral changes, including food consumption. Furthermore, studies examining the organ distribution of the materials, organ toxicities, hemocompatibility, and toxicokinetics are performed. The inflammatory responses of the organs are analyzed by measuring serum markers, including AST, ALT, LDH, BUN, etc. At the same time, the potential tissue damages are evaluated by the histopathological examination.^[^ ^20^ ^]^ In this study, the cell viability assays were performed on three different cell lines. The cell lines were selected based on the route of administration and target organs. These included the human vascular endothelial cell line (HUVEC), mouse fibroblast cell line (NIH/3T3), and mouse macrophage cell line (RAW264.7). The cells were treated with Ab‐MNPs (0–200 µg mL^−1^). The viability was measured by analyzing the metabolic activity of the live cells using a CCK‐8 assay. The results were presented as percentages compared with the control. No significant reduction of cell viability was observed until 200 µg mL^−1^ (**Figure** **4**a–c).

Hemocompatibility is a critical parameter for evaluating compound biocompatibility. Therefore, prior to in vivo administration, exposing nanoparticles to RBCs allows for the assessment of how the RBCs respond to the treatments. This exposure leads to disturbances in cell osmolarity, resulting in cell lysis, release of hemoglobin, and other consequences. Additionally, RBCs can agglutinate and fail to perform their normal functions.^[^ ^21^ ^]^ The hemocompatibility of Ab‐MNPs was evaluated in rodent blood. The negative and positive controls received PBS and distilled water, respectively. In contrast, the treated groups receive 25–200 µg mL^−1^ Ab‐MNPs. The results were presented as a percentage of hemolysis (Figure 4d). The observed hemolysis in Ab‐MNPs groups was <1%. Since it was <5%, based on hemocompatibility guidelines by ISO/TR 7406, it was hemocompatible.^[^ ^21^ ^]^

The organ distribution and biocompatibility of the AB‐MNPs were evaluated using an in vivo imaging system (IVIS). Fluorescently labeled anti‐CD3ε was conjugated with MNPs and administered at 10 mg kg^−1^ in BALB/C mice. The fluorescence distribution in major organs evaluated the distribution of the Ab‐MNPs after 0 h, 1 h, 1 day, and 7 days. The distribution of the nanoparticles conjugated with antibodies indicated the distribution pattern of the nanoparticles according to their size.

No detectable fluorescence was observed after 1 h. However, the fluorescence intensities increased after 24 h, and the highest fluorescence was observed after 7 days (Figure 4e). These findings indicate the significant metabolizing organ, the liver, stores the Ab‐MNPs after the kidneys. Further, the conjugated structure of Ab‐MNPs was stable as fluorescence movement followed the direction of the MNPs.

Toxicity was further evaluated in vivo through a single intravenous administration of Ab‐MNPs at the treatment dose (5 mg kg^−1^) and observation for 1 and 7 days. The mice were observed for feeding and toxicity behaviors each day afternoon. Mice were subsequently sacrificed, and samples (blood and organs) were collected and processed according to the experimental procedures. There were no abnormal behaviors observed during the experiment. The freshly collected wet organ weights were converted to organ indices by dividing corresponding mice weights and comparing them among the groups. The organ indices of day 1 and day seven mice were not significantly altered (Figure 4f). We also performed histology of the major organs to confirm acute tissue toxicity. The serum biomarkers related to liver, kidney, and broad‐spectrum tissue damage, including AST,^[^ ^22^ ^]^ BUN,^[^ ^23^ ^]^ and LDH,^[^ ^24^ ^]^ were measured using AU680 clinical chemistry analyzer (BECKMAN Coulter, Japan). The biomarker values were nonsignificant on day 1 and day 7 compared with control samples (Figure 4g–i). Furthermore, the hemolysis study assured that the Ab‐MNPs dose neither alters the RBCs' osmolarity nor agglutinates them. The biodistribution analysis also confirmed the stability of the conjugated Ab‐MNPs after 7 days of administration.

**Figure 5:**
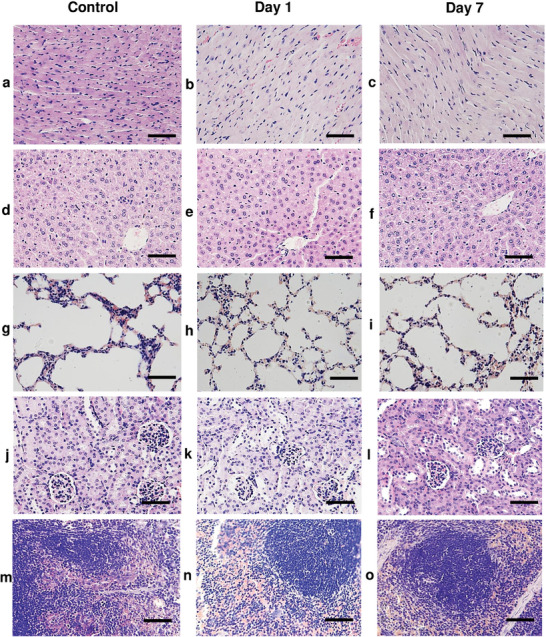
Histopathological analysis. Hematoxylin and eosin (H&E) staining was performed on the heart a–c), liver d–f), lung g–i), and kidney j–l), and spleen m–o) tissue sections. Mice were treated with Ab‐MNPs (5 mg kg^−1^), and organs were collected after 1 and 7 days. Organs from the untreated mice were used as control samples. The scale bar is 50 µm.

Furthermore, the histopathological study utilized major organs including the liver, kidney, lung, and heart. Following a single intravenous administration of Ab‐MNPs, the freshly collected organs were immediately fixed in a 4% paraformaldehyde solution. Hematoxylin and eosin (H&E) staining was then performed on 4 µm tissue sections. The observed heart (**Figure** **5**a–c), liver (Figure 5d–f), lungs (Figure 5g–i), kidney (Figure 5j–l), and spleen (Figure 5m–o) tissue samples were independently analyzed and confirmed no observable tissue damage in day 1 and day 7 mice compared with the control mice. Based on the in vitro cell viability and in vivo single‐dose treatment data, we confirmed no adverse signs of toxicity were observed. Therefore, the treatment dose of Ab‐MNPs is nontoxic and biocompatible.

### 2.5 Ab‐MNPs Decrease ConA‐Induced Increase in Serum Levels of Cytokines

**Figure 6:**
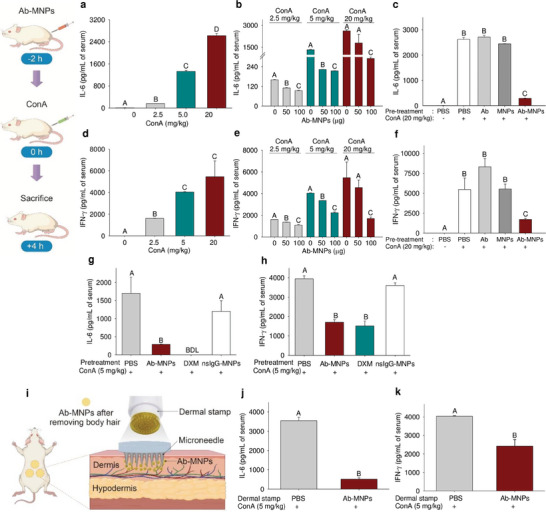
Attenuation of concanavalin A (ConA)‐induced inflammatory cytokines after pretreatment of Ab‐MNPs. ConA dose‐dependently induces interleukin (IL)−6 a) and interferon (IFN)‐γ d). Ab‐MNPs were pre‐administered (0, 50, and 100 µg) in low, medium, and high ConA‐treated mice. IL‐6 b) and IFN‐γ e) levels were quantified. The effects of Ab‐MNPs were validated by treating free MNPs and Ab c,f). Negative and positive control experiments were performed using non‐relevant IgG1 conjugated MNPs (nrIgG1‐MNPs) and dexamethasone (DXM) g,h) (*n* = 3 mice per group). Transdermal delivery of Ab‐MNPs using microneedle stamp patch. i) Usage of commercially available stamp patch (with 42 microneedles) of length 750 µm. Serum levels of IL‐6 j) and IFN‐γ k) in mice following stamp patch‐based delivery (*n* = 5 mice per group). *^A‐D^* Different letters indicate significant differences between groups (*p* < 0.05).

Monoclonal Abs (mAbs) used in immunotherapy are specifically designed to recognize antigens. In clinical practice, mAbs are primarily applied in disease diagnosis and cancer therapies.^[^ ^25^ ^]^ Moreover, Ab therapy has been used for autoimmune diseases, graft‐versus‐host disease, and allograft rejection.^[^ ^4^, ^26^ ^]^ Ab‐coupled nanoparticles recognize cellular targets, increase the therapeutic efficacy of drug candidates, and prevent toxicity. ConA is a plant lectin and potent mitogen that activates T cells and induces the secretion of proinflammatory cytokines.^[^ ^27^ ^]^ For this reason, ConA is frequently used to induce inflammation,^[^ ^28^ ^]^ acute liver injury,^[^ ^29^ ^]^ and autoimmune hepatitis.^[^ ^30^ ^]^ ConA activates T cells by an antigen‐independent mechanism. ConA binds with cell surface receptors, eventually activates the TCR‐CD3 complex, and induces T cells. Therefore, ConA does not activate T cells directly on CD3 receptors but rather indirectly activates TCRs. Anti‐CD3 mAbs (clone 145‐2C11) conjugated with dextran‐coated magnetite (Fe_3_O_4_) nanoparticles were designed to prevent the activation of the TCR–CD3 complex and Fc‐mediated interaction with APCs. Ab‐MNPs targeting the CD3ε chain of the TCR–CD3 complex were administered to female BALB/c mice (100 µL). The doses were calculated based on the concentration of MNPs and conjugated mAbs. The conjugated Ab‐MNP solutions (1 mL) comprised 1 mg of MNPs and 8 µg of mAbs. Therefore, 50 (50 µL) and 100 µg (100 µL) of conjugated MNPs in a mouse (average weight 20 g) represent MNP doses of 2.5 and 5 mg kg^−1^ and mAb doses of 0.02 and 0.04 mg kg^−1^, respectively. The intravenous injection of ConA dissolved in PBS at different concentrations (ensuring the administration of doses of 0, 2.5, 5, and 20 mg kg^−1^) increased the serum levels of interleukin 6 (IL‐6) (**Figure** **6**a) and interferon‐gamma (IFN‐γ) in a dose‐dependent manner (Figure 6d). Pretreatment of ConA‐injected mice with 100 µL of Ab‐MNPs (0.5 mg mL^−1^, 1 mg mL^−1^) resulted in a significant reduction in serum levels of IL‐6 (Figure 6b) and IFN‐γ (Figure 6e).

When low (2.5 mg kg^−1^) and medium (5 mg kg^−1^) doses of ConA were administered, the anti‐inflammatory effect of Ab‐MNPs was dose‐dependent. However, when a high ConA dose (20 mg kg^−1^) was used, a low dose of Ab‐MNPs (0.5 mg mL^−1^) did not prevent inflammation. Furthermore, MNP or anti‐CD3 mAb treatment alone did not counteract the inflammation induced by ConA (Figure 6c,f). Control treatments were performed with dexamethasone (DXM) and a nonrelevant IgG1(nrIgG1) conjugated with MNPs. After treatment with controls, the IL‐6 (Figure 6g) and IFN‐γ (Figure 6h) levels were quantified. The attenuation of cytokine levels with pretreatment of nrIgG1‐MNPs was insignificant compared to PBS treatment. The lowest and highest effective doses of HuM291 were 0.0015 and 0.015 mg kg^−1^, respectively.^[^ ^26b^ ^]^ Additionally, mAb doses of 0.02 and 0.04 mg kg^−1^ administered as conjugated Ab‐MNPs effectively reduced the levels of inflammatory cytokines. Concurrently, the doses of dextran‐coated MNPs administered were 2.5 and 5 mg kg^−1^, which are considerably below their reported safe dose of 10 mg kg^−1^.^[^ ^31^ ^]^

### 2.6 Microneedle‐Based Transdermal Delivery of Ab‐MNPs

The micro‐needling technique utilizes µm‐sized needles for nonpathogenic skin puncturing, stimulating skin cells to produce collagen and other growth factors.^[^ ^10^ ^]^ Initially, dermatologists used microneedles such as dermal rollers or stamps for antiaging treatment, reversal of skin wrinkles, and rejuvenation therapy.^[^ ^32^ ^]^ However, dermal rollers have recently been used in drug delivery.^[^ ^11^ ^]^ Several pharmaceutical formulations containing salicylic acid,^[^ ^33^ ^]^ nonsteroidal anti‐inflammatory drugs (NSAIDs),^[^ ^34^ ^]^ and protein drugs^[^ ^35^ ^]^ have been successfully delivered using these systems. Moreover, transcutaneous immunizations with dermal rollers resulted in improved responses. Thus, derma rollers increased the delivery of drug‐loaded liposome formulations two‐fold compared to passive delivery.^[^ ^36^ ^]^ Unlike dermal rollers, needles in a dermal stamp rest on a flat head of the dermal stamp. Therefore, dermal stamps are suitable for vertical skin penetration and drug administration over a small surface area of the skin.^[^ ^37^ ^]^ This study used a microneedle stamp patch (11.5 mm in diameter with 42 needles) for transdermal drug delivery. The titanium alloy‐based needles were 0.75 mm in length and were distributed at distances of 0.1 mm (Figure S2, Supporting Information). Considering the amount of Ab‐MNPs lost when using a needle stamp, we assumed that two‐thirds of the total volume of Ab‐MNP solution (150 µL; 1 mg mL^−1^) was administered to the dermis and subcutaneous tissue (Figure 6i). As shown in Figure 6j, the administration of Ab‐MNPs using a needle patch showed an 85% decrease in the concentration of ConA‐induced IL‐6, from 3500 to 500 pg mL^−1^. Similarly, the concentration of ConA‐induced IFN‐γ was reduced by 40%, from 4000 to 2400 pg mL^−1^ (Figure 6k).

**Figure 7:**
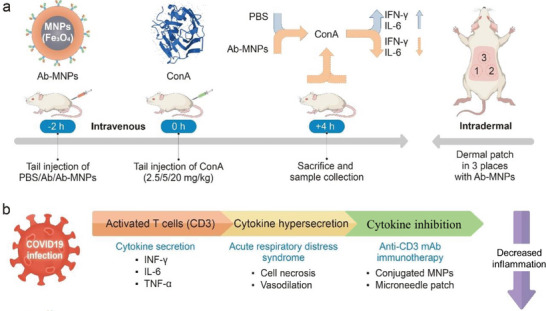
Schematic presentation of activated T cell‐mediated hyperinflammation and immunotherapy with Ab‐MNPs in a rodent inflammation model. a) Preadministration of Ab‐MNPs via intravenous administration and intradermal stamp patch‐based delivery inhibited concanavalin A (ConA)‐induced cytokine (IFN‐γ, IL‐6) hypersecretion. b) T cells are activated after viral infection and inflammatory cytokines are secreted. Cytokine hypersecretion, termed “cytokine storm,” is attenuated by anti‐CD3 monoclonal antibody‐conjugated magnetic nanoparticles (Ab‐MNPs).

Similar effects of Ab‐MNPs were obtained through dermal stamp patch delivery compared to intravenous administration through the tail vein. Accordingly, using different types of needle‐type patches could reduce the adverse effects of intravenous injection, while the efficacy would be similar. We found that both modes of administration inhibited ConA‐induced inflammation and reduced the serum levels of proinflammatory cytokines (**Figure** **7**a). We hypothesized that the newly synthesized complex can effectively inhibit T‐cell activation and reduce inflammation while avoiding major adverse effects (Figure 7b). This study suggests potential value in reducing T cell‐mediated hyperinflammation in organ transplantation and cytokine‐release syndrome in COVID‐19.

## 3 Conclusion

Targeted inhibition of the TCR component and tolerance induction are prerequisites for treatment with anti‐CD3 mAbs. The unavailability or modification of the Fc receptor of anti‐CD3 mAbs prevents Fc‐mediated interactions with APCs and induces further inflammation. In this study, amine‐functionalized dextran‐coated MNPs were successfully conjugated with anti‐CD3 mAbs using a simple strategy based on glutaraldehyde conjugation. Conjugation was performed to increase the bioavailability of anti‐CD3 mAbs and to prevent Fc‐mediated interactions. Data obtained from protein assays, immunocytochemistry, flow cytometry, fluorescence imaging, and electron microscopy validated the conjugation of anti‐CD3 with the MNPs. The hydrodynamic diameter of the MNPs increased owing to the attachment of Abs to their surface. The average observed diameter of MNPs and Ab‐MNPs was 45.0 and 51.06 nm, respectively. In the case of Ab‐MNPs, 90% of the particles increased in size from 54.7 ± 0.5 to 71.7 ± 2.7 nm. The in vitro and in vivo studies confirmed the therapeutic material as nontoxic and biocompatible. The preadministration of Ab‐MNPs reduced the serum levels of IFN‐γ and IL‐6 in a mouse model of ConA‐induced inflammation. However, treatment with MNPs and anti‐CD3 mAbs alone did not induce a significant anti‐inflammatory response. In addition, the effectiveness of the transdermal delivery of Ab‐MNPs using microneedle stamp patches was demonstrated in this study. Transdermal delivery also significantly reduced the serum levels of ConA‐induced IFN‐γ (45%) and IL‐6 (85%). The Ab‐MNPs bind with CD3 and induce modulation of the TCR‐CD3 complex.

Furthermore, the resultant T cells may become anergic or stimulate apoptosis. Therefore, our anti‐CD3 conjugated MNPs inhibit inflammation by inducing tolerance in T cells. The current study is valuable as it provides a potential solution for reducing T cell‐mediated severe inflammatory states in organ transplantation and cytokine‐releasing syndromes such as those observed in COVID‐19. Further, the CD3‐specific T cell activation model is promising in elucidating the mechanism of T cell inhibition.

## 4 Experimental Section

### Materials

Absolute Mag amine MNPs (dextran‐coated, 50 nm) were purchased from CD Bioparticles (NY, USA). Bovine serum albumin, glutaraldehyde solution (25%), ConA, DAPI, Tween 20, PepStrep, and Corning Costar TC‐treated multiple‐well plates were purchased from Sigma‐Aldrich Inc. (Saint Louis, MO, USA). RPMI 1640 cell culture media, 96‐well enzyme‐linked immunosorbent assay (ELISA) plates, 1× RBC lysis buffer, CD3ε monoclonal antibody (145‐2C11), NovaFlour700 tagged CD3ε monoclonal antibody, rabbit antihamster IgG (H+L), secondary antibody FITC labeled, and Pierce BCA Protein Assay Kit were purchased from Thermo Fisher Scientific (Waltham, MA, USA). Cell counting kit‐8 (CCK‐8) assay was purchased from Dojindo Laboratories Co. Ltd., Kumamoto, Japan. TEM copper grid support films, carbon 300 mesh, were purchased from Ted Pella, Inc. (Redding, CA, USA). The antifade mounting medium was purchased from Abcam (Cambridge, UK). Mouse IFN‐γ and IL‐6 ELISA kits were purchased from BD Biosciences (San Jose, CA, USA).

### Conjugation of Ab‐MNPs

Anti‐CD3 mAbs and amine‐functionalized dextran‐coated MNPs were conjugated by adding glutaraldehyde as the crosslinker. The two‐step conjugation process has been previously described.^[^ ^9^ ^]^ Briefly, MNPs (3 mg mL^−1^; 1 mL) dissolved in distilled water were washed several times and resuspended in PBS. The buffer separation/wash steps were performed by attaching plastic tubes containing MNPs to a permanent magnet (magnetic induction = 3.5 kG) for 12 h. Next, the MNPs were resuspended and mixed with glutaraldehyde solution (10%; 1 mL). Glutaraldehyde acts as a crosslinker that binds with amine groups on the surface of MNPs and amino acids (Arg, His, and Lys) of the Ab via an amide linkage. The mixture was then incubated at 25 °C with shaking. The glutaraldehyde‐functionalized MNPs were collected and washed several times to remove unbound glutaraldehyde. The glutaraldehyde‐functionalized MNPs were stored at 4 °C until further use. Next, anti‐CD3 mAb solution (200 µg mL^−1^; 100 µL) was mixed with glutaraldehyde‐functionalized MNPs (3 mg mL^−1^; 333 µL) and PBS (567 µL). The solution was mixed thoroughly and incubated at 4 °C for 12 h. The Ab‐MNPs were collected and washed five times to remove unbound Abs. Finally, BCA protein assays were performed to measure free and conjugated Abs levels. The resultant Ab‐MNPs were stored at 4 °C until further use. The conjugated nanoparticles were further validated using a UV–vis spectrophotometer, immunocytochemistry, fluorescence imaging, and flow cytometry.

### Nanoparticle Analysis by Field Emission‐Transmission Electron Microscope (FE‐TEM) and Scanning Electron Microscope (SEM)

FE‐TEM was employed to capture the nanoscale features of nanoparticles in 2D images and analyze their elements. The MNPs and Ab‐MNPs were loaded onto a TEM copper grid with a carbon 300 mesh. SEM was used to analyze and obtain 3D images of the particles and EDS was performed on the local and total areas of the particles to determine their elemental composition.

### NTA of MNPs and Ab‐MNPs

The size distribution of MNPs and Ab‐MNPs was determined using NTA with a NanoSight NS300 system (Malvern Panalytical Technologies, Worcestershire, UK). The equipment included a scientific metal‐oxide‐semiconductor sensor‐based camera, 20× objective lenses, green laser modules, and NTA software version 3.4. The capture settings were as follows: level, 16; slide shutter, 1300; slide gain, 295; number of frames, 1498; and number of frames per second, 25. Samples were diluted to an acceptable concentration in particle‐free PBS and delivered using a 1 mL disposable syringe. The video data for NTA measurements were collected for 30 s and repeated five times for each sample. The detection threshold of NTA software was set to five, and the maximum jump distance and minimum track segment length were set to auto.^[^ ^38^ ^]^

### Immunocytochemistry Analysis

The splenocytes were immunostained with either anti‐CD3 or Ab‐MNPs (MNP‐conjugated anti‐CD3 Ab). The mouse splenocyte isolation method has been previously described.^[^ ^39^ ^]^ First, splenocytes were fixed in 100% methanol (chilled at −20 °C) for 10 min. Next, the cells were incubated with anti‐CD3 Ab in a blocking buffer (1× PBS with 0.1% Triton X‐100) overnight at 4 °C. After washing with Tris‐buffered saline (150 mm NaCl, 50 mm Tris‐HCL, pH 7.6), the cells were incubated with secondary Abs in a blocking buffer for 2 h at 25 °C. After staining with DAPI, coverslips were mounted, and images were obtained at 40× and 100× magnifications using a fluorescence microscope.

### Flow Cytometry Analysis

Splenocytes collected from immediately sacrificed mice were counted, and 5 × 10^5^ cells mL^−1^ were stained. Anti‐CD3 antibodies labeled with NovaFlour700 fluorescence were added to the Ab group. Fluorescent anti‐CD3 antibodies conjugated with MNPs (Ab‐MNPs) were added to the Ab‐MNPs group. Unstained or negative controls were used for proper compensation. Cells were washed thrice in PBS supplemented with 10% fetal bovine serum and 0.1% sodium azide. Samples were kept in a chilled buffer and analyzed on the next day. Samples were run on a Gallios instrument (Beckman Coulter Inc., Indianapolis, IN, USA) using a red channel (FL7 detector) that excites at 638 nm and emits at 725 nm. The samples were analyzed using Kaluza analysis software.

### In Vitro Toxicity Evaluation

In vitro, cell viability evaluations were performed in human HUVEC, NIH/3T3, and RAW/264.7 cell lines. Confluent cells were incubated with Ab‐MNPs at 0–200 µg mL^−1^ in a 96‐well plate. The CCK‐8 measures the viability of cells by analyzing their ability to convert water‐soluble tetrazolium salt to formazone. The resultant orange‐colored formazone was measured at 450 nm. The data were presented as a percentage of proliferation compared with the control sample.

### Animal Experiments

BALB/c mice (three to five mice/group; seven to eight weeks old, female, 18–22 g) were purchased from DBL Korea (Chungcheong‐buk, Korea). The mice were acclimatized for 1 week in the laboratory (12 h dark/light cycle, humidity 50–60%, and temperature 25 °C). The mice were fed a routine laboratory diet and provided water ad libitum. All experimental procedures were performed according to the local regulations and approved by the Animal Ethics Committee (IACUC) of Sangji University, Korea, according to the Animal Experiment Regulations (No. 2022–01).

### In Vivo Toxicity Studies

Seven‐ to eight‐week‐old female BALB/c mice were randomly divided into three groups. Mice were administered intravenously once with an Ab‐MNPs suspension similar to the treatment dose (5 mg kg^−1^) or PBS solution. Animals were observed daily for body‐weight changes and clinical signs of toxicities, including tremors, ataxia, and mortality. Mice were sacrificed, and samples were collected on day 1 and 7 after treatment.

### In Vivo Toxicity Studies: Serum Collection Biochemical Analysis

Blood samples were collected via cardiac puncture after anesthesia with an injection of Zoletil‐Rompun. Serum was collected by leaving the blood at 25 °C for 1 h and centrifuging at 2000 × *g* for 10 min. The sera were aliquoted and stored at −80 °C until analysis. Serum biochemical marker for hepatic function was evaluated by measuring alanine aminotransferase (AST). Lactate dehydrogenase (LDH) was measured to assess tissue damage, and kidney function was measured by measuring blood urea nitrogen (BUN).

### In Vivo Toxicity Studies: Histopathological Analysis

After collecting blood samples, the major organs, including the heart, liver, lungs, kidneys, and spleen, were collected and fixed in 4% paraformaldehyde solution (pH 7.4). Tissues were then embedded in paraffin blocks, sectioned (4 µm) using Leica RM2145 rotary microtome (Leica Biosystems, Wetzlar, Germany), and attached to a glass slide. Slides were kept on a slide warmer for 24 h and stained with H&E dyes. Histopathological analysis was performed by an expert pathologist at 400× magnification using a light microscope.

### Biodistribution of Ab‐MNPs Using IVIS

Mice were intravenously administered 200 µg (10 mg kg^−1^ of body weight) of MNP‐Ab‐NovaFlour700 in 100 µL PBS. The ex vivo organ (liver, kidneys, spleen, heart, lungs, and thymus) distributions of the flourescent Ab‐MNPs were measured after 1 h, 1 day, and 7 days using the FOBI in vivo imaging system (Cellgentek, Daejeon, Korea). The near‐infrared (NIR) color channel captured the fluorescence signal from the freshly collected mouse organs.

### RBC Hemolysis

A hemolysis study was performed with freshly collected blood from mice. In brief, fresh blood was collected in a tube containing 5% EDTA. Blood was centrifuged thrice at 2000 × *g* for 5 min each in PBS, with 50 µL of RBC used for each treatment. Negative control (0% lysis) and positive samples (100% lysis) were incubated with PBS and distilled water. Ab‐MNPs were added in triplicate in 25, 50, 100, and 200 µg mL^−1^. Samples were mixed and incubated for 1 h at 25 °C. Samples were then centrifuged at 5000 rpm for 5 min. The supernatant samples were distributed in a 96‐well plate and measured at 540 nm in a spectrophotometer. The hemolysis rate was calculated as:

(1) $$\mathrm{Hemolysis}\;\left(\%\right)=\left(\left(D_{t}-D_{\mathrm{nc}}\right)/\left(D_{\mathrm{pc}}-D_{\mathrm{nc}}\right)\right)\times 100$$

where, *D*_t_, *D*_nc_, and *D*_pc_ are the absorbance of tested samples, negative and positive control, respectively.

### ConA‐Induced Inflammation Mice Model

The mice were treated with low, medium, and high doses (2.5, 5, and 20 mg kg^−1^, respectively) of ConA dissolved in PBS via intravenous tail injection to induce considerable inflammation. Control mice were treated with only PBS. Two hours before ConA injection, the mice were pretreated with Abs, MNPs, or Ab‐MNPs via intravenous tail injection. Finally, 4 h after the ConA injection, the mice were sacrificed, and blood samples were collected.

### Transdermal Delivery of Ab‐MNPs

After anesthesia, the hair on the dorsal skin of the mice was removed from three regions selected for drug delivery. Fifty microliters of Ab‐MNP‐containing (1 mg mL^−1^) solution was given in two stamps; the Ab‐MNP solution (25 µL) was dropped on the skin surface at each site and administered through the skin surface by applying force with a needle stamp, administering Ab‐MNPs (100 µg). After 2 h, ConA (5 mg kg^−1^) was injected into the tail vein to induce inflammation. After 4 h, the levels of cytokines in the collected serum were measured using ELISA.

### Quantification of Cytokines

Cytokine (IFN‐γ and IL‐6) levels in the serum samples were measured using ELISA protocols provided by the manufacturers. Briefly, 96‐well plates were coated with capture Abs and incubated overnight at 4 °C. After washing, the wells were blocked with assay diluents. The plates were then incubated with standards and samples. After washing, the plates were incubated with a secondary Ab conjugated to horseradish peroxidase before adding 3,3′,5,5′‐tetramethylbenzidine substrate solution. Color formation was measured at 450 nm using an Infinite pro‐2000 microplate reader (Tecan Group Ltd., Manndorf, Switzerland). Measurements were made in duplicate. The results are presented as pg mL^−1^ of serum sample.

### Statistical Analysis

The data are expressed as mean ± standard error of the mean. Comparisons between the two groups were made using Student's *t‐*test. The one‐way analysis of variance (ANOVA) was used to compare multiple groups. Statistical analysis and figures are prepared with SigmaPlot 10.0 from SPSS Inc. (Chicago, IL, USA).

### Ethics Approval

Mouse dissection experiments were approved by the Animal Ethics Committee (IACUC) of Sangji University, Korea, according to the Animal Experiment Regulations (No. 2022–01).

## Conflict of Interest

The authors declare no conflict of interest.

## Supporting information

Supporting Information

## Data Availability

The data that support the findings of this study are available in the supplementary material of this article.
