# Supplementary material for: Antibody‐Conjugated Magnetic Nanoparticle Therapy for Inhibiting T‐Cell Mediated Inflammation
Source: Adv Sci (Weinh). 2023 Dec 31;11(11):2307148. doi: 10.1002/advs.202307148 (PMC10953552; doi:10.1002/advs.202307148)
Supplement: Supplementary file 1 — Supporting Information [file ADVS-11-2307148-s001.pdf]

ADVANCED SCIENCE
Open Access

Supporting Information

for *Adv. Sci.*, DOI 10.1002/advs.202307148

Antibody-Conjugated Magnetic Nanoparticle Therapy for Inhibiting T-Cell Mediated Inflammation

*Mahbub Hasan, Jong-Gu Choi, Hafeza Akter, Hasung Kang, Meejung Ahn and Sang-Suk Lee**

Supporting Information

Antibody-Conjugated Magnetic Nanoparticle Therapy for Inhibiting T-cell Mediated Inflammation

*Mahbub Hasan[1,2], Jong-Gu Choi[1], Hafeza Akter[1], Hasung Kang[3], Meejung Ahn[4,] Sang-Suk Lee[1]**

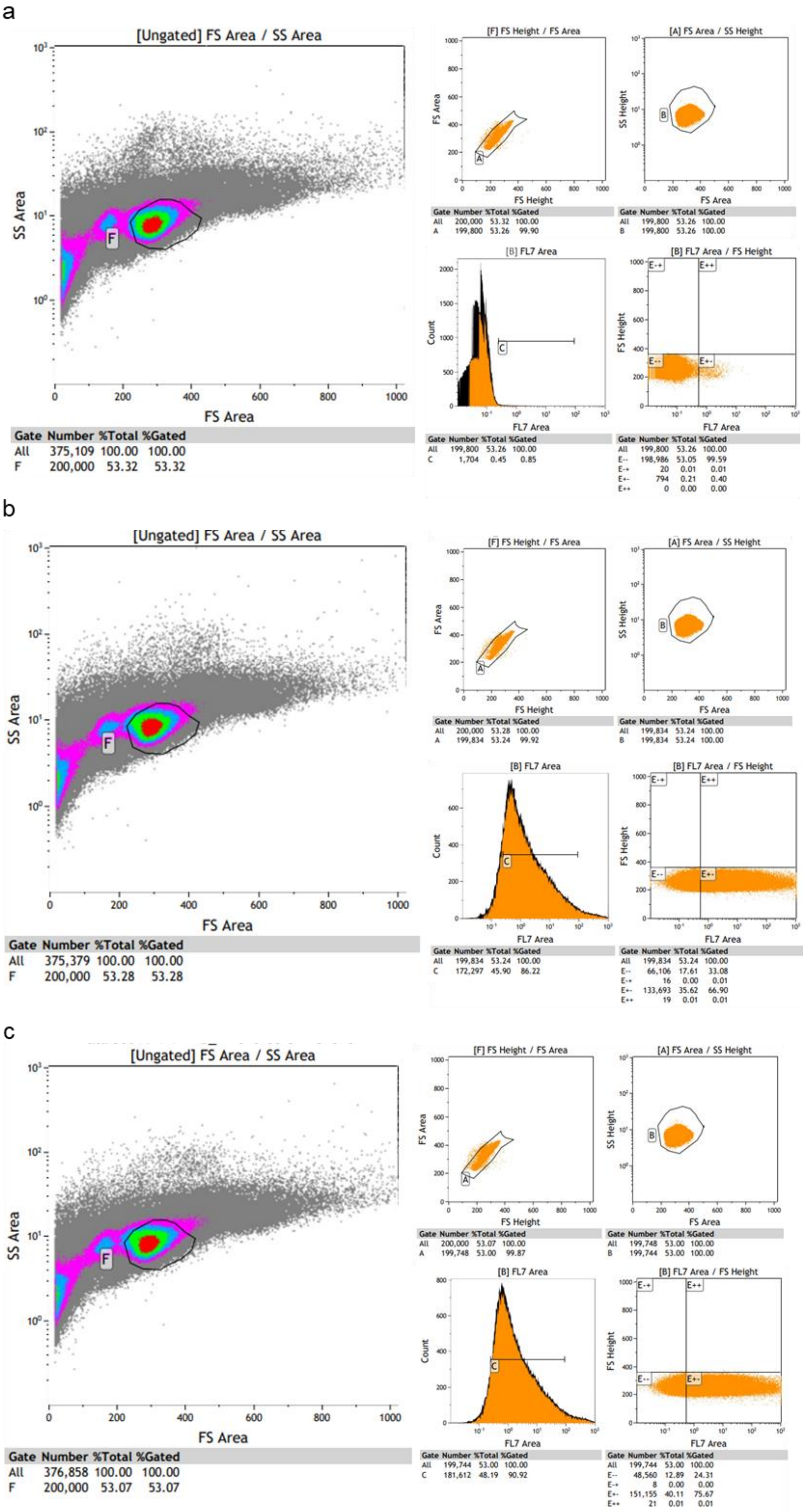

Figure S1: Flow cytometric analysis of CD3-positive cells. The gating of CD3-positive cells for unstained control (a), Ab-Novaflour700 (b) and Ab-Novaflour-MNPs (c) are presented.

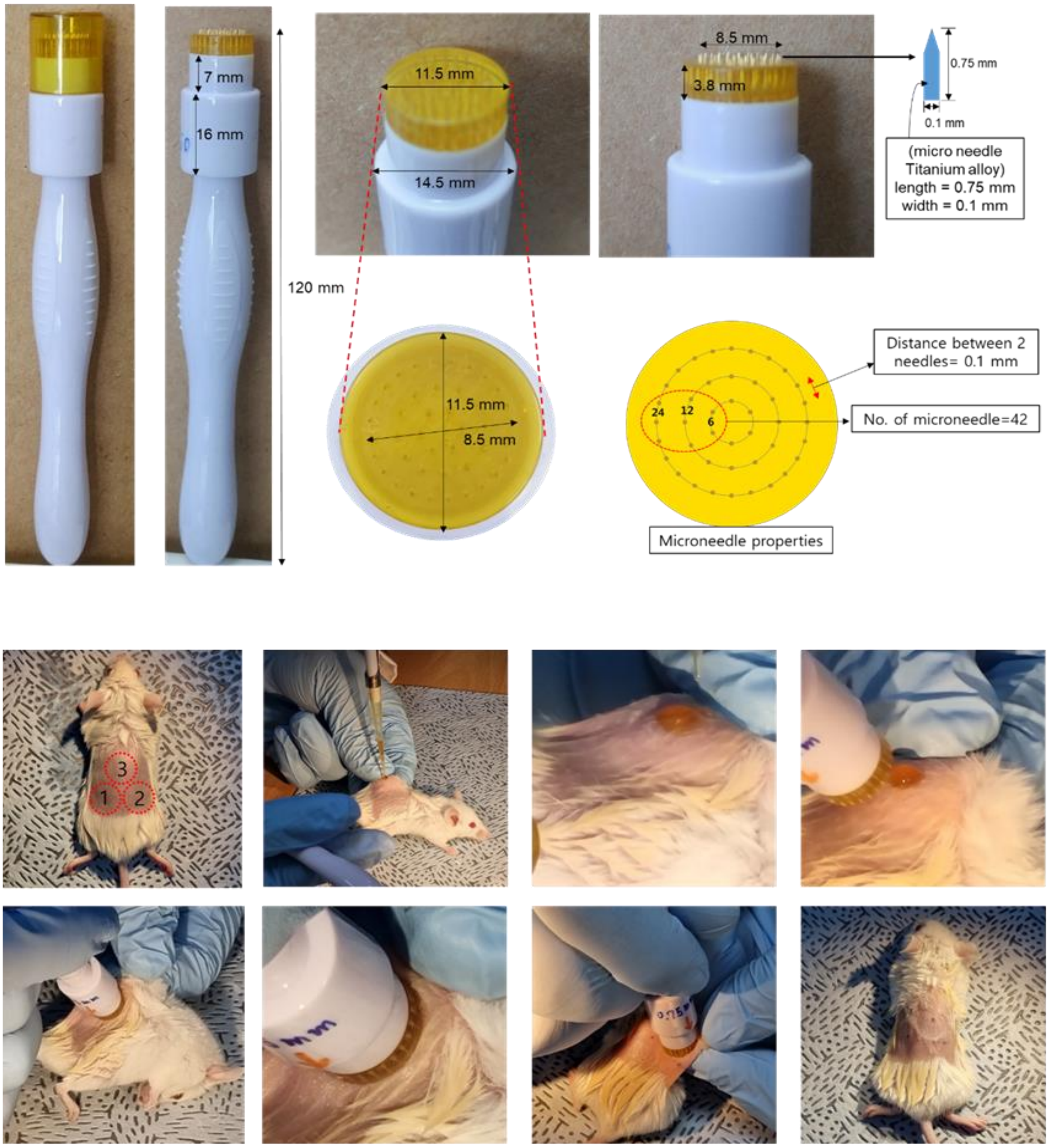

Figure S2: Properties of stamp patch and drug delivery procedure in mice. Commercially available stamp patch (with 42 microneedles) of length 750 µm. After anesthesia, the hair on the dorsal skin of a *BALB/c* mouse was removed, and three regions were selected for stamp patch treatment. In total, 150 µL(3 regions × 50 µL) of Ab-MNPs was applied to each mouse.
